# Automated Segmentation and Severity Analysis of Subdural Hematoma for Patients with Traumatic Brain Injuries

**DOI:** 10.3390/diagnostics10100773

**Published:** 2020-09-30

**Authors:** Negar Farzaneh, Craig A. Williamson, Cheng Jiang, Ashok Srinivasan, Jayapalli R. Bapuraj, Jonathan Gryak, Kayvan Najarian, S. M. Reza Soroushmehr

**Affiliations:** 1 Department of Computational Medicine and Bioinformatics, University of Michigan, Ann Arbor, MI 48109, USA; gryakj@umich.edu (J.G.); kayvan@umich.edu (K.N.); ssoroush@umich.edu (S.M.R.S.); 2 Michigan Center for Integrative Research in Critical Care (MCIRCC), University of Michigan, Ann Arbor, MI 48109, USA; craigaw@med.umich.edu; 3 Department of Neurological Surgery and Neurology, University of Michigan, Ann Arbor, MI 48109, USA; 4 Department of Neurology, University of Michigan, Ann Arbor, MI 48109, USA; 5 Department of Electrical Engineering and Computer Science, University of Michigan, Ann Arbor, MI 48109, USA; chengjia@umich.edu; 6 Department of Radiology, University of Michigan, Ann Arbor, MI 48109, USA; ashoks@med.umich.edu (A.S.); jrajiv@med.umich.edu (J.R.B.); 7 Michigan Institute for Data Science (MIDAS), University of Michigan, Ann Arbor, MI 48109, USA; 8 Department of Emergency Medicine, University of Michigan, Ann Arbor, MI 48109, USA

**Keywords:** clinical decision support system, subdural hematoma, medical image processing, machine learning, deep learning

## Abstract

Detection and severity assessment of subdural hematoma is a major step in the evaluation of traumatic brain injuries. This is a retrospective study of 110 computed tomography (CT) scans from patients admitted to the Michigan Medicine Neurological Intensive Care Unit or Emergency Department. A machine learning pipeline was developed to segment and assess the severity of subdural hematoma. First, the probability of each point belonging to the hematoma region was determined using a combination of hand-crafted and deep features. This probability provided the initial state of the segmentation. Next, a 3D post-processing model was applied to evolve the initial state and delineate the hematoma. The recall, precision, and Dice similarity coefficient of the proposed segmentation method were 78.61%, 76.12%, and 75.35%, respectively, for the entire population. The Dice similarity coefficient was 79.97% for clinically significant hematomas, which compared favorably to an inter-rater Dice similarity coefficient. In volume-based severity analysis, the proposed model yielded an F1, recall, and specificity of 98.22%, 98.81%, and 92.31%, respectively, in detecting moderate and severe subdural hematomas based on hematoma volume. These results show that the combination of classical image processing and deep learning can outperform deep learning only methods to achieve greater average performance and robustness. Such a system can aid critical care physicians in reducing time to intervention and thereby improve long-term patient outcomes.

## 1. Introduction

Trauma is the leading cause of death among Americans younger than 46 [1]. Traumatic brain injuries (TBI), which contribute substantially to traumatic mortality and morbidity, are frequently referred to as the silent epidemic [2]. Each year, about 1.7 million people sustain TBI in the U.S., among whom approximately 52,000 die, while 126,000 survivors experience long-term impairment or disability [2]. One-third of patients who died because of TBI were able to talk or obey commands before their death. This suggests that the effect of secondary injuries, rather than the initial injuries at the time of trauma, is an important contributor to mortality [3]. Thus, the early diagnosis and management of TBI, particularly during the “golden hour”, the period of time following the incident, could significantly minimize mortality and the progression of secondary injuries. 

In this paper, we focus on the diagnosis and evaluation of subdural hematoma (SDH), which is one of the most common types of traumatic intracranial hemorrhage encountered in neurosurgical practice. SDH refers to the accumulation of blood in the potential space between the arachnoid mater surrounding the brain and below the skull and dura mater. As the blood accumulates in the subdural region, it can compress the underlying brain parenchyma and lead to focal neurological deficits, unconsciousness, and death. Therefore, the existence of SDH may require emergency treatment by surgically evacuating the blood. 

The total volume of hematoma is an important factor in the diagnosis and prognosis of TBI patients [4,5,6,7]. For instance, the widely accepted Marshall scale for TBI severity rating and treatment planning considers the hematoma volume as one of its few key metrics [5]. However, manual measurement is time consuming and almost impossible to implement in practice. Automated image analysis could rapidly measure total hematoma volume and quantify other clinically relevant measurements that are not typically available from a conventional review. Moreover, an automated diagnosis system enables earlier detection, thereby alerting the radiologists for higher prioritization of the imaging study. However, automated detection of SDHs can be challenging due to variability in size, location, and brightness/intensity observed in a head Computed Tomography (CT) scan. The intensity of blood varies substantially over time depending on chronicity of SDH, leading to three main types: acute, subacute, and chronic. Acute hematoma is most frequently encountered following a severe head injury and in a head CT scan, it is identified by its relative brightness (intensity) compared to normal brain tissue. Subacute hematoma develops over weeks, with its brightness similar to normal brain tissue. Chronic hematoma tends to occur in elderly patients with brain atrophy, often as a result of minimal trauma occurring weeks or even months before presentation and looks darker than normal brain tissue on CT scans. 

Automated imaging analysis can act as a safeguard or triage tool for alerting radiologists and clinicians of potentially moderate and severe injuries. Additionally, automated analysis can also quickly measure clinically relevant image features that are difficult to quantify by conventional visual inspection. In the case of SDH, guidelines for surgical intervention and many treatment decisions are based on simple measurements of maximum hematoma width at a single CT slice. Hematoma volume is not typically measured or reported because accurate measurement is difficult and time-consuming. Automated image analysis could rapidly measure total hematoma volume and quantify other clinically relevant measurements that are not typically available from a conventional review. Since subdural hematomas frequently expand and sometimes require multiple surgical interventions, another application could be using quantitative volumetric measurements to accurately assess both progression of disease and response to surgical intervention. 

Consequently, the goal of this study was to design an automated platform to detect SDH and assess its severity by measuring its volume in patients with potential TBI. Moreover, we performed an inter-physician variability analysis to determine a human performance benchmark. The resulting algorithm could enable timely intervention, thereby improving survival while lowering the risk of secondary injuries. 

For the SDH segmentation task, classical image processing approaches were integrated with a deep convolutional neural network model to overcome the limitations of each method. The proposed algorithm employs a joint feature representation of domain knowledge-driven hand-crafted features and data-driven deep features to train a random forest model.

## 2. Related Works

To the best of our knowledge, there is no prior published work, except our preliminary study [8], that describes automated methods to detect and segment the different types of SDH. However, there are several techniques developed for acute hematoma detection/segmentation, which are reviewed here. These techniques fall into semi- or fully-automated categories regarding human interaction. These segmentation techniques either implement traditional rule based methods [9,10,11,12,13] or employ machine learning methods [14,15,16,17,18]. Yuh et al. [9] evaluate the presence or absence of acute intracranial blood from CT scans in an automated fashion. Their findings include the presence or absence of: (1) SDH or epidural hematoma, (2) subarachnoid hemorrhage, and (3) intraparenchymal hematoma. First, regions with an intensity similar to blood are detected using thresholding. Then, the detected potential region is categorized into one of the above three categories based on its location, size, and shape. If a blood cluster is contiguous to the skull, it is defined to be SDH or an epidural hematoma. Otherwise, it is further divided into subarachnoid or intraparenchymal based on shape and location. However, this study only accounts for acute hematoma. Liao et al. [10] focus on hematoma in different brain locations. In their method, all patient images with subacute (with the same brightness as normal brain tissue) and/or chronic (darker than normal brain tissue) hematoma regions are manually excluded. For other images, they focus on a single pre-selected CT slice containing the largest intracranial area. Within that slice, the largest hyperdense component is found. Next, a level set method is applied to evolve the segmentation. In a more recent study [11], the same research group proposed a multiresolution binary level set method to identify hematomas in patients with neurological disorders. As in their previous work, only acute hematomas were considered. Moreover, all of the included CT images were from patients who underwent brain surgery. These restrictions resulted in selected slices having a large portion of blood, making detection less challenging. Chan et al. [12] introduced a method to segment small acute brain hematomas. This method first detects bright objects by simple thresholding and then searches for right-left asymmetry of brain tissue to select candidates. Finally, acute hematoma regions are identified based on both anatomical location and image features. The proposed symmetry analysis, however, is highly dependent on the fact that axial slices are perfectly parallel to the axial plane, which is not always the case. The method proposed in Li et al. [13] aims to segment acute intracranial hemorrhage. In this method, an information gain algorithm is implemented to adaptively determine the hematoma intensity interval. In this model, it is assumed that all the images have a hematoma, negating the need to identify non-hematoma images. 

A fuzzy C-means (FCM) clustering and active contour model were employed in Bhadauria et al. [14] to segment acute hematoma. First, FCM was used to group the brain tissue based on image intensity. An active contour model was then applied to refine the contours of the clusters detected by FCM. The number of clusters in the FCM algorithm must be predefined; however, this number is subject to change based on the presence/absence of hematoma and also other types of TBIs. In Shahangian et al. [15], a fixed threshold was applied to images to approximate acute hemorrhage regions. Then, multiple textural and geometrical features were extracted from a selected region to classify it as intracranial, epidural hematoma, or SDH. This work was further improved in Shahangian et al. [16] by applying an adaptive threshold instead of a fixed value. There are a few research studies related to brain hematoma detection that employ convolutional neural networks. The focus of [17,18] was to determine the presence or absence of acute hematomas in CT images without investigating the hematoma segmentation and severity assessment. 

One problem with most of the aforementioned studies is that they narrowly focus on anatomic location and the size of the hematoma. For instance, only a hematoma thickness larger than 4 mm is considered in Liao et al. [10]. Additionally, none of the referenced studies focus on different types of hematomas, even though subacute and chronic SDHs are widely presented in specific patient populations, such as in the elderly, who frequently present with a combination of acute and chronic hematomas. Moreover, for the rule-based models, algorithmic hyperparameters are often manually selected by analyzing the whole dataset, rather than just the training set, potentially producing a model overfitted to the particular dataset. Additionally, except for Bardera et al. [19], no other work takes advantage of available 3D information. The use of 2D images alone fails to consider spatial coherency that can help avoid false positive or false negative regions.

In our preliminary work [8], we reported on 35 CT scans that were included in the current study. The current study expands on this by including a much larger sample size, substantially improving the segmentation algorithm by integrating domain knowledge into data-driven deep models, performing further analyses, and comparing performance with a human benchmark.

## 3. Materials and Methods

### 3.1. Patient Population

This HIPAA Compliant, IRB-approved (HUM00118873) retrospective study utilized a dataset of TBI patients aged 18 years or older admitted to the University of Michigan Neurological Intensive Care Unit (ICU) or Emergency Department from 1 January 2010 to 10 August 2015. In this study, written informed consent from patients was waived by IRB because the research involved no more than minimal risk to the subjects. The only risk to subjects is breach of confidentiality and loss of privacy. Safeguards were in place to prevent this from happening. Moreover, research could not practically be carried out without the waiver or alteration because this study exclusively involved the secondary analysis of information that was collected as a part of routine medical care. A number of patients to be included in the study could have died or been lost to follow-up so could not conceivably be contacted to provide consent. We included 98 consecutive patients with SDH at the cerebral convexities as well as 12 subjects with normal head CT scans. We did not include interfalcine hematomas in the midline.

### 3.2. CT Image Settings

All the images used to develop our method were acquired in the axial plane on 64 slice CT scanners (either LightSpeed VCT or Discovery CT HD750, GE Healthcare Milwaukee, WI, USA). Images were stored on a McKesson Picture Archiving and Communication System (PACS). All CT scans were acquired with 0.625 mm thickness and reformatted to 5 mm slice thickness for storage purpose while the pixel spacing in the axial plane ranged from 0.4297 mm to 0.4883 mm. A medium filter was inserted into the X-ray beam for image reconstruction in all the CT scans.

### 3.3. CT Image Annotation

In order to train our model, we used images annotated by two skilled fellowship trained neuroradiologists with 29 and 16 years of experience, respectively. The “Free Hand” tool of the MicroDicom DICOM viewer software was employed to annotate the CT scans [20]. Both neuroradiologists were blinded to other information. Although annotating images from multiple experts could minimize inter-physician variability and thereby improve label accuracy, performing this process is labor-intensive and prevents the creation of a large training set. Therefore, in our study, the dataset was divided into two parts and each part was annotated by one of the neuroradiologists. All annotations were then reviewed and adjudicated by an expert board-certified physician in neurology and neurocritical care. If any annotation done by one of the neuroradiologists appeared to include a potential human error (as adjudicated by the neuro ICU physician), the other neuroradiologist was asked to review and revise the annotation if indicated. In this way, annotation uniformity was optimized and improved. The adjudicated annotations are used as the ground truth throughout the paper. A subset of 20 CT scans was annotated by both neuroradiologists to determine the inter-physician variability.

### 3.4. Experimental Design

**Figure 1:**
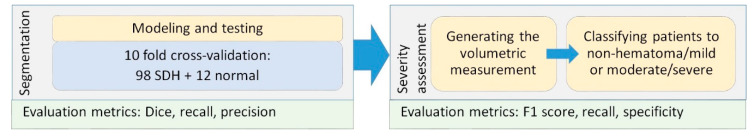
Experimental design and evaluation strategy for subdural hematoma (SDH) segmentation and severity assessment.

The study design for SDH segmentation and severity assessment are shown in Figure 1. We first developed a machine learning model using 10 fold cross validation to segment SDH regions. Then, we employed the automatically segmented region to assess the severity of SDH.

### 3.5. Machine Learning Pipeline

**Figure 2:**
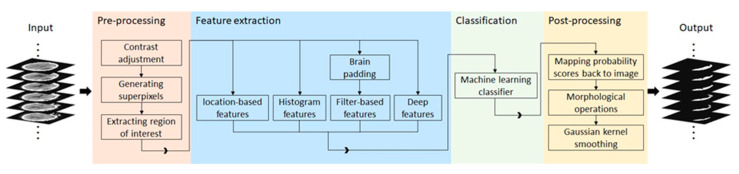
A schematic diagram of the proposed SDH segmentation method.

Figure 2 demonstrates a high-level overview of the proposed method. Given a head CT scan, we first performed pre-processing to identify the region of interest (ROI). Next, potential discriminative patterns were extracted from the ROI, followed by using a random forest classifier to identify SDH regions. Finally, in the post-processing step, morphological operations and Gaussian kernel smoothing were used to improve overall segmentation performance.

#### 3.5.1. Pre-Processing

Pre-processing began with the generation of a 3D representation of the CT slices from a sequence of 2D images and was followed by intensity normalization of images according to the provided metadata, skull segmentation, and intracranial segmentation, similar to our previously proposed pre-processing method [8]. Then, intracranial pixels (i.e., pixels enclosed by the skull) were grouped into superpixels, which were used throughout the feature extraction and classification steps.

##### Extracting the Region of Interest

In our database, even the most severe case of SDH was not deeper than 3.2 cm from the skull. Therefore, the ROI was defined as the intracranial region within 3.2 cm of the inner skull. A level-set method was employed to segment the intracranial region enclosed by the skull in case there were any openings in the skull boundary [8]. These openings can exist due to either normal anatomy such as the eye cavity or traumatic injury such as a fracture. If the skull was closed in the axial plane, simply, all pixels enclosed by it were selected as the intracranial region. More information can be found in our previous work [8].

##### Sampling

Once the ROI was delineated, the image was divided into non-overlapping regions of connected pixels with approximately similar gray value. Superpixels were used instead of pixels to reduce redundant information. In this work, we used the simple linear iterative clustering (SLIC) algorithm [21] for generating superpixels.

#### 3.5.2. Feature Extraction

Feature extraction was performed to derive patterns and statistics that represent superpixels. These features were later used as inputs to the machine learning classifiers to discriminate SDH superpixels from non-SDH ones. In this study, we considered hand-crafted textural and spatial features, as well as deep image features. Additionally, the patient′s age is incorporated as an auxiliary variable into the machine learning algorithms. Age-related brain atrophy predisposes patients to chronic subdural hematoma formation and can also lead to subdural hygroma, the accumulation of cerebrospinal fluid within the skull, which can be mistaken for chronic SDH. A full list of extracted features can be found in Table A1 (Appendix A). 

**Figure 3:**
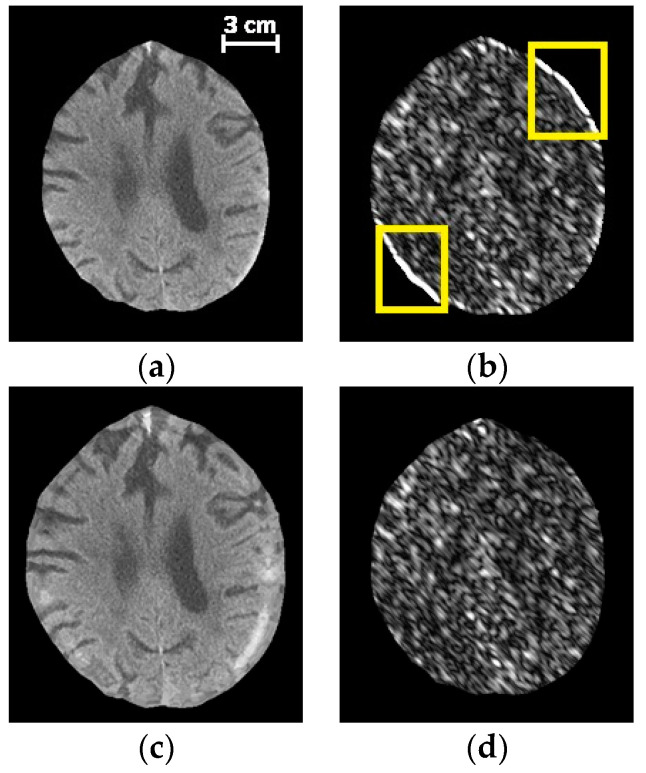
The effect of padding on a Gabor descriptor. (**a**) The original image before padding, (**b**) the magnitude response for a Gabor filter at $\frac{3\pi }{4}$ (**c**) the image after applying the proposed padding method, (**d**) the magnitude response for the Gabor filter at $\frac{3\pi }{4}$ applied on (**c**).

Histogram and filter analyses were performed to extract hand-crafted local textural information from the images. In order to derive local appearance information, a window with a fixed size of 25 × 25 pixels was localized around the center of each superpixel from which the corresponding features were derived. However, if the window was centered on superpixels close to the skull, where SDH tends to occur, the selected window would include pixels from skull. Thus, extracted textural features from the window would be affected by skull pixels and might not correctly represent SDH characteristics. For example, features that normally represent the orientation of brain/hematoma texture, would instead represent the skull orientation, which is not informative. Since SDH tends to occur adjacent to the skull, extracting correct features in this region is especially important for our purposes. In order to deal with this challenge, we removed the skull from the image and padded the image with a symmetric mirror reflection across the inner surface of the skull. As the boundary of the brain is not a straight line, we could not employ standard padding techniques. Instead, we proposed to iteratively pad the image across the irregular border of the intracranial region. This task was performed by maintaining two masks: the inner mask that shrank in each iteration and the outer mask that grew in each iteration. The region grown during each iteration was filled using the nearest pixel on the inner mask. Figure 3 illustrates the intracranial region before and after padding, as well as the corresponding filtered image by applying a Gabor filter. The used Gabor filter highlights the textural component in the $\frac{3\pi }{4}$ orientation. In Figure 3b, though there is no significant textural component in the $\frac{3\pi }{4}$ orientations, the filter enhances the border incorrectly within the region enclosed in yellow. As shown in Figure 3d, this issue was resolved using the proposed padding method.

##### Histogram-Based Statistical Features

For each superpixel, a histogram of pixel intensity inside the corresponding window was generated. The histogram-derived features included minimum, maximum, average, and standard deviation, σ, of pixel intensity. The average intensity of pixels within the superpixel was also calculated. Other features were skewness, kurtosis, entropy, and smoothness. There are various definitions of “smoothness”. In our approach, smoothness was calculated using Equation (1).

(1) $$smoothness=1-\;\frac{1}{1-\;\sigma^{2}}$$

##### Filtering-Based Features

To integrate more textural information, a group of features was extracted by convolving Gabor and Laplacian of Gaussian filters with the images. Gabor filters were used to extract image features at multiple orientations and frequencies. The Gabor filter was calculated by Equation (2), where *u*_0_ is the frequency of a sinusoidal carrier along the *x*-axis and *σ_x_* and *σ_y_* are, respectively, the constant of the Gaussian envelope along the *x* and *y* axes. To build the filter for orientations other than 0°, a rigid rotation of the *x*-*y* coordinate was performed [22].

(2) $$h\left(x,\;y\right)=exp\left(-\;\frac{1}{2}\;\;\left[\frac{x^{2}}{\sigma_{x}^{2}}\;+\;\frac{y^{2}}{\sigma_{y}^{2}}\right]\right)\;\cdot \;\cos\left(2\pi u_{0}x\right)$$

In this study, we used Gabor filters in 8 evenly spaced orientations {0, $\frac{\pi }{8}$, …, $\frac{7\pi }{8}$}, each at 4 wavelengths of the sinusoidal carrier, ranging from 2 to 16 pixels/cycle. 

The Laplacian of Gaussian filter determines the edge content of the images in a localized region. This filter measures the second spatial derivative of a smoothed image and highlights the edges. Gaussian filters with five different kernels were implemented to smooth the image.

##### Location-Based Features

**Figure 4:**
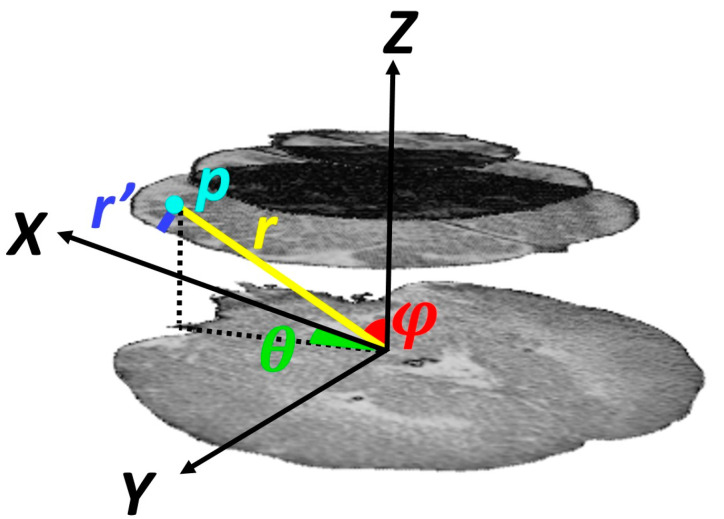
Illustration of location-based features of point *p* within the region of interest.

As mentioned above, subdural hematomas are more likely to occur in specific regions. Thus, location information was incorporated into our model by extracting features in a spherical coordinate system. These parameters are radial distance, azimuth angle, and elevation angle. The origin was fixed on the center of the mass of the skull on the lowest selected slice. As shown in Figure 4, the elevation angle, *φ*, determines the angle between the horizontal plane and the line connecting the point to the origin. The azimuth angle, *θ*, corresponds to the angle between the projection of the line connecting the point to the origin on the horizontal plane and the brain midline that separates it into two hemispheres. The radial distance, *r*, measures the distance of the point to the origin. As proximity of a point to the skull increases the likelihood of injury, in addition to the radial distance, we considered the distance between the point and the inner surface of the skull, *r*′.

##### Deep Features

**Figure 5:**
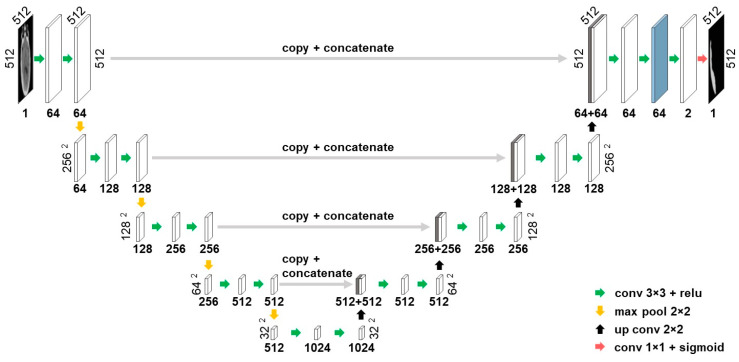
Illustration of the baseline U-net architecture used for segmentation. The activations of the layer marked in blue are used as deep features.

In this work, the U-net architecture was employed to extract data-driven deep features. The U-net architecture was proposed by Ronneberger et al. [23] in 2015 and has subsequently become one of the most widely used convolutional neural network architectures for biomedical applications, specifically those with limited access to annotated data. First, a baseline U-net model shown in Figure 5 was trained for SDH segmentation and then activations of the second layer before the output layer were employed as deep features. The layer used for deep feature extraction is marked by blue in Figure 5 and yields 64 features for each superpixel. To ensure that deep features were not influenced by the test set, *n*-fold cross-validation was performed, in which *n* different models generated *n* sets deep features. Later, the same cross-validation folds were used to train the random forest model.

#### 3.5.3. Classification

Once the aforementioned features were extracted for each superpixel, a random forest model was trained to classify each superpixel sample as hematoma or non-hematoma. As the number of negative sample points (superpixels) was approximately eight times more than positive sample points, the classes were highly imbalanced. To overcome this challenge, undersampling on the negative samples was performed, in which all positive points were kept and an equal number of points from the negative class were randomly selected to be used in the training phase.

#### 3.5.4. Post-Processing

Using the label calculated for each superpixel in the classification step, the initial SDH mask is formed. However, this initial SDH mask was generated without accounting for information in neighboring pixels. 

Multiple disjoint subdural hematomas may occur simultaneously. However, pixels within each distinct hematoma must be connected. This contextual information was ignored during the previous superpixel classification step. As such, there might be misclassified superpixels that can be corrected by considering their neighboring regions. In order to resolve this issue, small sparse components were first excluded from the initial 2D SDH mask. Next, morphological operations were applied to fill the small holes and gaps between the segmented mask and the skull. Additionally, 2D masks disconnected within the third dimension were excluded. In addition to applying morphological operations, the segmented mask is filtered using a 3D Gaussian smoothing kernel to smooth jagged contours and increase spatial coherency.

### 3.6. Hematoma Volumetric Measurement for Severity Assessment

Hematoma volume is a key metric used in TBI severity ratings, such as the widely used Marshall scale [5]. However, because its manual measurement is not easy, subdural hematoma width rather than total volume is much more routinely used in clinical practice. For the purposes of severity assessment, we categorized hematoma severity based upon volume: <25 cc—non-hematoma/mild; and ≥25 cc—moderate/severe. To quantify the hematoma volume, we used the segmented SDH regions as a binary mask and calculated the volume of the lesion using the CT slice resolution from the CT scan metadata.

### 3.7. Inter-Physician Variability Analysis

To create a benchmark with which to evaluate segmentation performance, we sought to determine the inter-physician variability of subdural hematoma annotation. In this analysis, a set of 20 SDH patients representing the same volumetric distribution as the original 98 patients were selected. We asked two skilled neuroradiologists to independently annotate the selected set. The Dice similarity coefficient was used to compare the two sets of annotations.

## 4. Results

### 4.1. Classification Result

To train the classifiers, the dataset of 110 patients was partitioned into 10 folds for cross-validation. The folds were generated to include a roughly balanced distribution of SDH types. The random forest yielded the average area under the receiver operating characteristic curve (AUC) of 0.9777 in classifying superpixels using the combination of hand-crafted and deep features.

### 4.2. Subdural Hematoma Segmentation

Table 1 compares the segmented SDH region with the ground truth. The Dice similarity coefficient (Dice) is a summary measure of spatial overlap between the segmented region and the ground truth. Dice, sensitivity, precision, and specificity evaluation metrics are defined as

(3) $$\mathrm{Dice}\;=\;\frac{2\;\times \left|S\;\cap \;GT\right|}{\left|S\right|+\left|GT\right|}\;\mathrm{Recall}\;=\;\;\frac{\left|S\;\cap \;GT\right|}{\left|GT\right|}\;\mathrm{Precision}\;=\;\frac{\left|S\;\cap^{\;}\;GT\right|}{\left|S\right|}\;\mathrm{Specificity}\;=\;\frac{\left|\bar{S}\;\cap \;\bar{GT}\right|}{\left|\bar{GT}\right|}$$

where *GT* and *S* refer to the manually annotated ground truth and the algorithm segmentation, respectively. | . | denotes set cardinality and $\bar{A}$ is the complement of *A*.

Table 1 shows that applying post-processing increased the average Dice and decreased its variation. Since there was no SDH and hence, no ground truth region annotated for patients without SDH, Dice, recall, and precision values were not defined for such subjects and therefore they are not included in the table. For these subjects, specificity was calculated to determine what portion of negative pixels within the defined ROI was correctly classified. For healthy subjects, our algorithm reached an average specificity (±68% standard error of the mean) value of 99.89 (±0.10)%.

**Figure 6:**
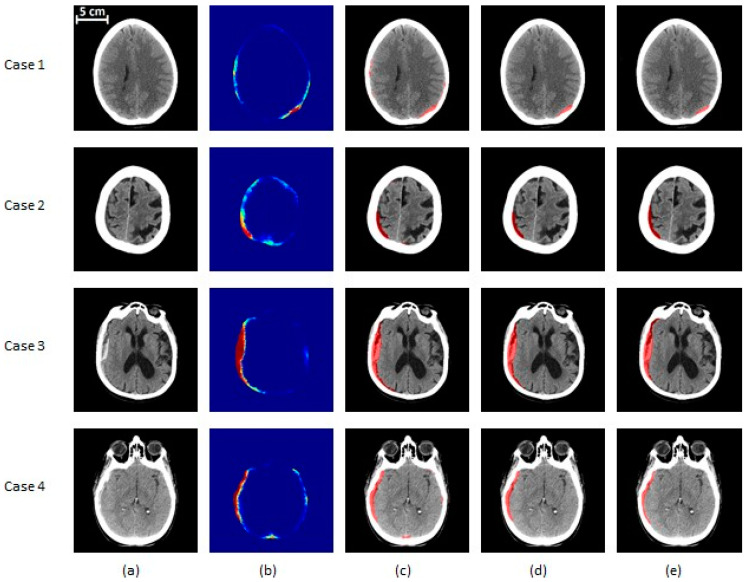
SDH segmentation results on mild and moderate subjects. (**a**) The original CT image, (**b**) the probability map corresponding to the output of the classifier, (**c**) the segmented region before post-processing, (**d**) the segmentation result after post-processing, (**e**) the ground truth. Cases 1 and 2 are mild (<25 cc of blood) patients and cases 3 and 4 are moderate (25–50 cc of blood) ones. Cases 1, 3, and 4 are acute SDH, while case 2 is an example of a chronic hematoma. In (**a**,**c**–**e**), the gray region corresponds to brain texture in the original CT image. In (**b**), the colormap ranging from blue to red is associated with probability from zero to one.

**Figure 7:**
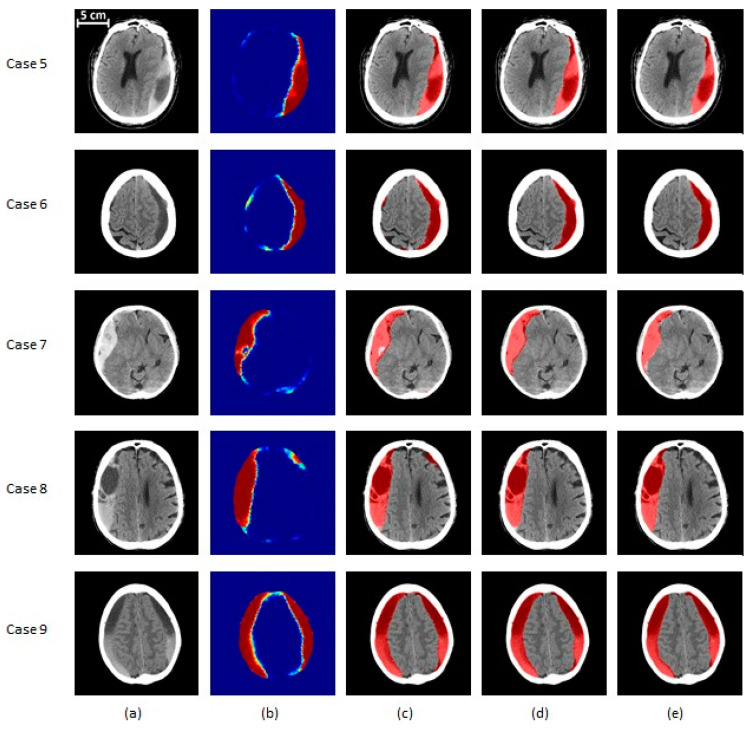
SDH segmentation results on severe (>50 cc of blood) subjects. (**a**) The original CT image, (**b**) the probability map corresponding to the output of the classifier, (**c**) the segmented region before post-processing, (**d**) the segmentation result after post-processing, (**e**) the ground truth. Cases 5 and 6 are severe patients with total blood volume of less than 100 cc, while cases 7 and 8 have total blood volume of 100 to 200 cc. Finally, case 9’s SDH volume is over 200 cc. Cases 5, 8, and 9 contain a mix of acute and chronic hematoma. Case 6 is an example of chronic hematoma, while case 7 is acute. In (**a**,**c**–**e**), the gray region corresponds to brain texture in the original CT image. In (**b**), the colormap ranging from blue to red is associated with probability from zero to one.

The results of the proposed segmentation algorithm at different stages are shown in Figure 6 and Figure 7. These images cover various combinations of size and type of subdural hematoma.

**Figure 8:**
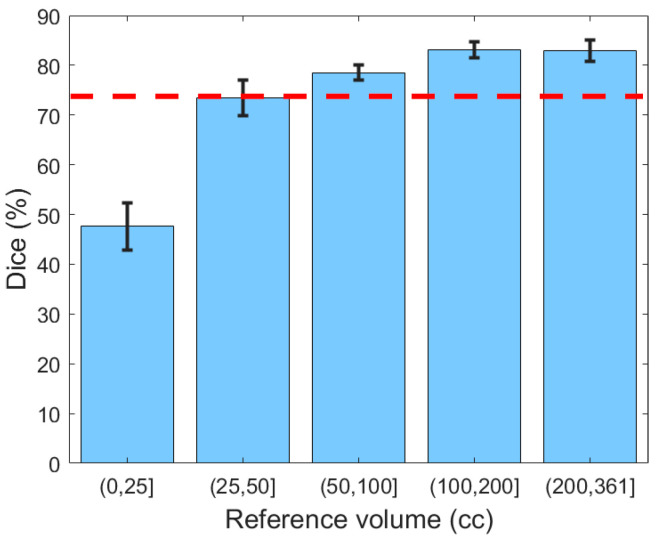
The Dice similarity coefficient based on the severity of hematoma. Error bars represent ±1 standard errors, the 68% confidence interval. The red dashed line indicates the human benchmark.

Next, the generalizability of the proposed segmentation approach was further validated by analyzing segmentation performance with respect to different ranges of hematoma volume (Figure 8). The reference volume was calculated using the ground truth and was discretized by thresholding at 25, 50, 100, and 200 cc. It can be concluded from Figure 8 that Dice values for subjects with mild SDH (47.67%) were lower than for those with more severe SDH (79.97%), possibly because the smaller regions were less represented in the classifier. Moreover, for small lesions, even a small deviation in segmentation has a large impact on the Dice similarity value.

**Figure 9:**
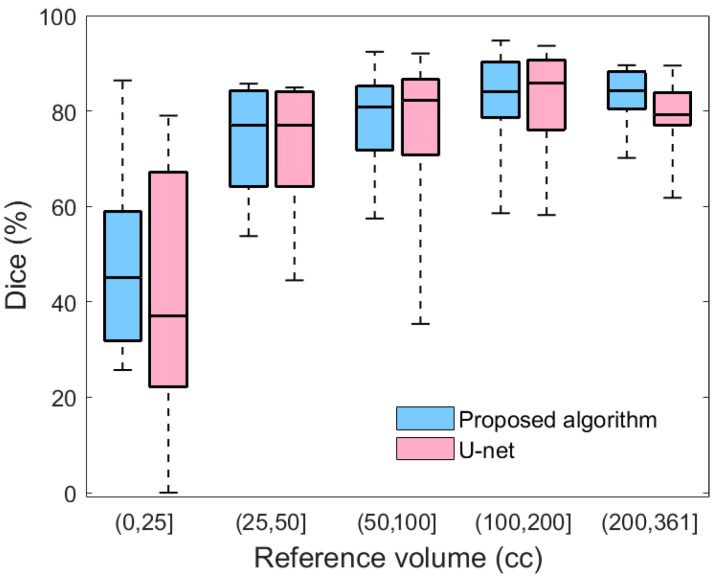
A box plot comparing the Dice similarity coefficients of the proposed algorithm and U-net with respect to the severity of hematoma.

Table 1 also provides a comparison between the performance of the proposed method and the U-net model. To ensure a fair comparison, both models were trained using 10-fold cross-validation. As shown in Table 1, the proposed method outperforms U-net in terms of both the average Dice value and the standard error of the mean. Moreover, the summary statistics in Figure 9 evidence that compared to U-net, the proposed method yields less variability for all severity levels. Likewise, the lower extreme of the proposed method is greater than the lower extreme of U-net in all categories. In particular, for mild hematomas with less than 25 cc of blood, more than 25% of U-net’s Dice values are lower than any of those from the proposed method. The lower average performance and higher variability of U-net may be because deep learning approaches require a large and representative sample of annotated images. Thus, a deep learning model trained on limited datasets may fail to reflect the unseen spectrum of the real-world data distribution. Integrating hand-crafted features that reflect human domain knowledge can compensate for this limitation of deep models, yielding segmentation results that are more consistent and robust.

### 4.3. Generalizability of the Segmentation Model over Different Types of SDH

We investigated the performance of the proposed algorithm with respect to subdural hematoma type (Table 2). We used the metrics defined in Equation (3) to compare the algorithm’s segmentation results with the manually annotated ground truths.

**Figure 10:**
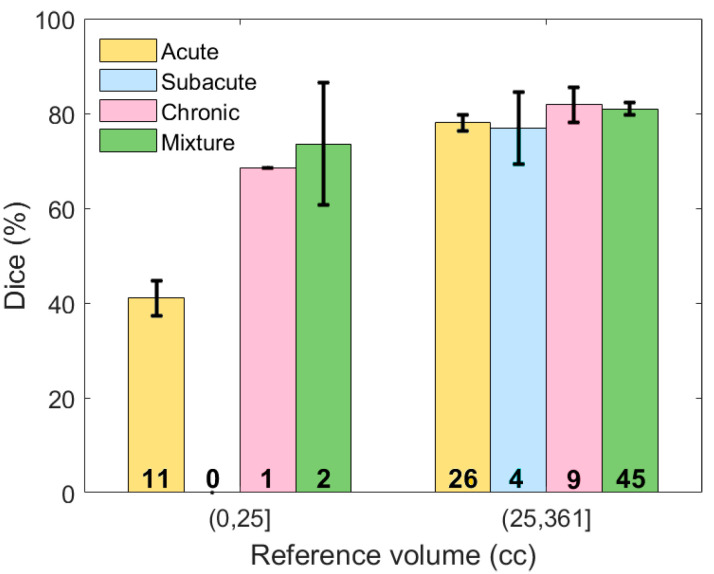
The Dice similarity coefficient based on both the severity and type of hematoma. Error bars represent ±1 standard errors, the 68% confidence interval. The number of samples for each category is shown on the corresponding bar.

Based on Table 2, it might be concluded that the overall performance on subjects with acute SDH was lower than the other three types. However, chronic and mixed subdural hematomas tend to be larger in elderly patients because there is more space for the hematoma to expand without symptoms due to brain atrophy. Thus, the proportion of the subjects with acute SDH and less than 25 cc of blood is higher than other types and as discussed earlier, these small hematoma regions are more challenging to detect. Thus, in order to have a valid comparison, the subjects were first grouped according to volume, after which the performance was compared for different types. Figure 10 shows the average Dice similarity coefficient for each category. Based on this plot, for moderate and severe SDH, the performance of the proposed segmentation algorithm was consistent among different subdural types. We do not have a big enough sample size to either reject or accept performance consistency among different types of the mild (<25 cc) SDH subjects.

### 4.4. Inter-Physician Variability Analysis

**Figure 11:**
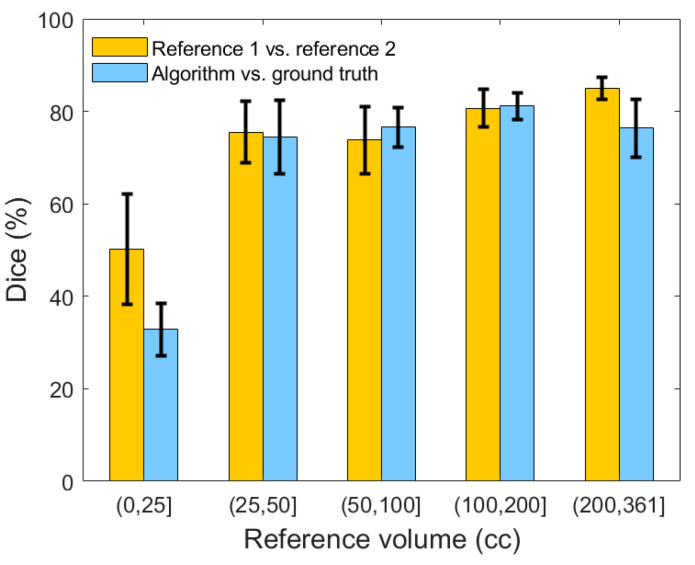
Comparison of the Dice similarity coefficient between reference 1, reference 2, and the result of our segmentation algorithm. Error bars represent ±1 standard errors, the 68% confidence interval.

Figure 11 illustrates the comparison between the two neuroradiologists, as well as a comparison of our algorithm’s segmentation against the adjudicated ground truth with respect to the selected 20 patients. The average inter-rater Dice similarity coefficient on the selected subset of 20 SDH CT scans is 73.71%, while this coefficient is 50.20% and 77.85%, respectively, when stratifying lesions based on <25 cc and ≥25 cc of blood. On the same subset, the algorithm′s average Dice similarity coefficient value reached 77.81% for moderate and severe SDHs compared to 77.85% for human raters.

### 4.5. Hematoma Volume Assessment for Severity Analysis

**Figure 12:**
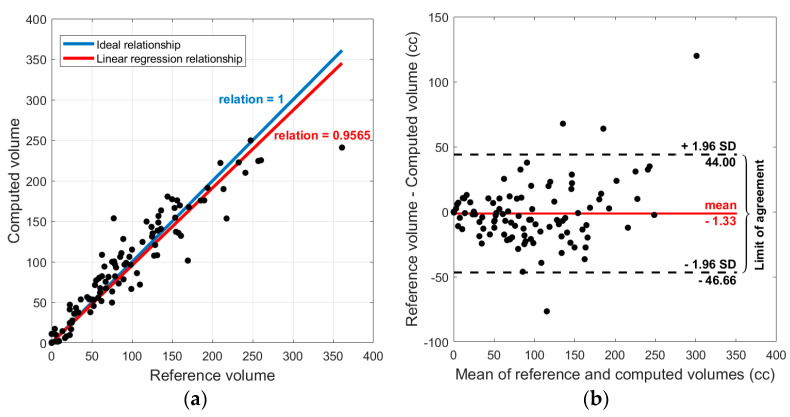
(**a**) Linear regression relation between the computed volume and reference volume. Each point corresponds to one patient. (**b**) Bland-Altman plot, which indicates the normality of error.

Total hematoma volume was measured for all patients and then used to stratify hematomas by severity. Figure 12a illustrates a linear regression between the volume resulting from the algorithm and the ground truth volume for 110 studied patients. From this plot, it can be concluded that the algorithm tends to under-segment larger lesions while it overestimates the smaller ones. The linear regression relation is 0.96 (SE 0.02, *p*-value < 0.01). 

Next, Bland-Altman analysis was performed to determine the agreement between the reference and computed volumetric measurements. Figure 12b shows the corresponding Bland-Altman plot. This result shows a bias close to zero: −1.33 cc (95% confidence interval −46.44 to 44.00), suggesting that there is no systematic error in calculating SDH volume using the algorithm.

One of the applications of this analysis was to develop tools that will alert medical providers when a patient requires immediate intervention, which in the context of subdural hematoma, corresponds to the ability to identify volumetrically large hematomas. Table 3 shows the confusion matrix in classifying patients according to severity based on their lesion volume as defined above. The recall, specificity, and F1 score in detecting moderate/severe patients (more than 25 cc of SDH volume) are 98.81%, 92.31%, and 98.22%, respectively.

## 5. Discussion

In this study, we proposed a fully automated approach for segmentation and radiographic severity assessment of subdural hematoma at cerebral convexities by analyzing head CT scans. By developing a machine learning technique (i.e., a classifier) to divide brain tissue pixels to SDH and non-SDH followed by applying a post-processing step, we performed the SDH segmentation and achieved an average Dice similarity coefficient of 75.35% in segmenting subdural hematoma. The Dice similarity coefficient was 79.97% for moderate and severe SDHs. Our segmentation algorithm reached an average specificity value of 99.89% for healthy subjects, which indicates the algorithm’s high performance in avoiding false positive pixels in healthy subjects. The model proved to be generalizable to different types of SDHs with more than 25 cc of blood. Besides evaluating the method by using Dice, precision, and recall, we performed a Bland-Altman analysis to see if there is any systematic error or bias in the computed volumetric measurement and found neither of these. Based on the automatically measured volume of hematoma, we categorized SDH patients to severe and non-severe ones and achieved the recall, specificity, and F1 score of 98.81%, 92.31%, and 98.22%, respectively, in detecting moderate or severe subjects. However, we should acknowledge that the control cases are from patients with no brain injuries and do not include scans with non-SDH mass lesions. For the population of interest, moderate and severe hematoma cases (i.e., more than 25 cc of SDH), our model was shown to be robust in segmentation of all types of SDH. This model enables the accurate quantification of blood, which otherwise is almost impossible due to the time-consuming manual process. 

Importantly, the results show that compared to deep learning models, integrating descriptive hand-crafted features with data-driven deep features leads to a higher overall accuracy as well as greater consistency and robustness in segmenting SDH regions. The primary reason is that the hand-crafted features reflect human domain knowledge, which can compensate for the limitations in deep model performance on unobserved regions of the input data distribution. 

In our inter-physician variability study to create a human benchmark, we created two independent sets of ground truth from a representative subset of CT scans. We showed that there is 73.71% agreement between the ground truths created by two skilled neuroradiologists. This finding indicates that there is uncertainty in the gold standard (i.e., manual) labels. This issue is due to the fact that in lesion studies, including hematoma detection, the border between the affected region and the adjacent healthy brain is not necessarily well-separated. Using inaccurate labels to train a machine learning algorithm can adversely affect its performance as well.

To our knowledge, no prior study has previously described automated methods to identify the different SDH types. Given the significant variations in size and intensity of SDHs in CT scans, developing a generalizable algorithm is challenging, but essential because many patients (in particular, the elderly) present with a combination of both acute and chronic hematomas. In this work, we sought to address these challenges by proposing a learning algorithm that employs the advantages of both classical image processing and deep learning approaches.

Although the results are comparable with the human benchmark, there are limitations in this study. One of the limitations is the lack of enough samples to investigate the generalizability of the algorithm over different types of blood for mild SDHs. This reflects the pathophysiological mechanism of this type of traumatic injury. Chronic and mixed SDH are more prevalent in the elderly population for whom there is more space inside the skull for the hematoma to expand before experiencing symptoms. Another limitation is that the model is trained on anisotropic resolution images, with low resolution retrieved from PACS. PACS down-samples the images along the longitudinal axis while storing them, which can reduce the image resolution. For instance, the pixel spacing along the longitudinal axis after saving them could be over 10 times greater than the spacing along the sagittal and frontal axes (e.g., 5 mm vs. less than 0.5 mm). This anisotropic resolution could lead to discontinuity along the longitudinal axis after down-sampling, hence affecting the recall of the algorithm specifically for small hematoma regions. The proposed model was trained on CT scans captured using LightSpeed VCT or Discovery CT750 HD systems, both from GE, the U-M vendor of choice. CT scans from other manufacturers could be incorporated into future models to create a vendor-agnostic model. 

Even though quantitative CT characteristics improve diagnosis and outcome prediction, traditional visual examinations are still the only investigation done by clinicians, which are qualitative rather than quantitative, prone to human error, and costly. The automated detection and quantitative measurement of moderate and severe SDH could provide a basis to improve diagnostic accuracy and to prevent delayed diagnosis. Thus, the ability to accurately detect and quantify larger and therefore more clinically significant subdural hematomas can facilitate timely recognition of the patients in greatest need of early treatment interventions. In this study, automated SDH segmentation and assessment models were developed, which demonstrated a robust performance comparable to the human benchmark.

Regarding the future direction of this work, the proposed algorithm should be tested and validated against a larger population from an external data set and obtain regulatory approval and clinician acceptance before it can be incorporated into clinical practice. The predictive power of the SDH volumetric measurement should also be evaluated against a representative data set that covers different variations of SDH in traumatic brain injury patients.

## Figures and Tables

**Table 1:** A comparison of the mean of the segmentation performance metrics before and after post-processing as well as the performance of the baseline U-net architecture. Numbers in parentheses are 68% standard error of the mean.

Performance Metric	Proposed Method without Post-Processing	Proposed Method	U-Net
Dice (%)	73.63 (±1.64)	75.35 (±1.58)	73.20 (±2.02)
Recall (%)	80.72 (±1.69)	78.61 (±1.93)	70.93 (±2.15)
Precision (%)	70.75 (±1.76)	76.12 (±1.52)	79.61 (±2.03)

**Table 2:** Mean of the segmentation performance metrics for acute, subacute, and chronic types and their mixture. Numbers in parentheses are 68% of standard error of the mean.

Performance Metric	Acute (*n* = 37)	Subacute (*n* = 4)	Chronic (*n* = 10)	Mixture (*n* = 47)
Dice (%)	67.05 (±3.24)	76.93 (±7.67)	80.54 (±3.56)	80.66 (±1.30)
Recall (%)	73.00 (±4.13)	75.59 (±12.63)	83.49 (±4.65)	82.26 (±1.76)
Precision (%)	68.88 (±2.98)	81.20 (±2.49)	78.60 (±3.48)	80.85 (±1.63)

**Table 3:** Confusion matrix for classifying lesion severity by volume.

Reference\Calculated	0–25 cc	>25 cc
0–25 cc	24	2
>25 cc	1	83

## Appendix A

**Table A1:** The comprehensive list of the utilized hand-crafted and deep features.

Feature Type	Feature/Feature Class	
Domain knowledge-driven	Age	
	Location-based	Radial distance
		Azimuth angle
		Elevation angle
		Distance to skull
	Histogram-based	Minimum
		Maximum
		Average
		Standard deviation
		Skewness
		Kurtosis
		Entropy
	Filtering-based	Gabor
		Laplacian of Gaussian
Data-driven	Deep features	
